# A Broad-Spectrum Chemokine-Binding Protein of Bovine Papular Stomatitis Virus Inhibits Neutrophil and Monocyte Infiltration in Inflammatory and Wound Models of Mouse Skin

**DOI:** 10.1371/journal.pone.0168007

**Published:** 2016-12-09

**Authors:** Saeed Sharif, Yoshio Nakatani, Lyn Wise, Michael Corbett, Nicola C. Real, Gabriella S. Stuart, Zabeen Lateef, Kurt Krause, Andrew A. Mercer, Stephen B. Fleming

**Affiliations:** 1 Department of Microbiology and Immunology, University of Otago, Dunedin, New Zealand; 2 Department of Biochemistry, University of Otago, Dunedin, New Zealand; Katholieke Universiteit Leuven Rega Institute for Medical Research, BELGIUM

## Abstract

Bovine papular stomatitis virus (BPSV) is a *Parapoxvirus* that induces acute pustular skin lesions in cattle and is transmissible to humans. Previous studies have shown that BPSV encodes a distinctive chemokine-binding protein (CBP). Chemokines are critically involved in the trafficking of immune cells to sites of inflammation and infected tissue, suggesting that the CBP plays a role in immune evasion by preventing immune cells reaching sites of infection. We hypothesised that the BPSV-CBP binds a wide range of inflammatory chemokines particularly those involved in BPSV skin infection, and inhibits the recruitment of immune cells from the blood into inflamed skin. Molecular analysis of the purified protein revealed that the BPSV-CBP is a homodimeric polypeptide with a MW of 82.4 kDa whilst a comprehensive screen of inflammatory chemokines by surface plasmon resonance showed high-affinity binding to a range of chemokines within the CXC, CC and XC subfamilies. Structural analysis of BPSV-CBP, based on the crystal structure of orf virus CBP, provided a probable explanation for these chemokine specificities at a molecular level. Functional analysis of the BPSV-CBP using transwell migration assays demonstrated that it potently inhibited chemotaxis of murine neutrophils and monocytes in response to CXCL1, CXCL2 as well as CCL2, CCL3 and CCL5 chemokines. In order to examine the effects of CBP *in vivo*, we used murine skin models to determine its impact on inflammatory cell recruitment such as that observed during BPSV infection. Intradermal injection of BPSV-CBP blocked the influx of neutrophils and monocytes in murine skin in which inflammation was induced with lipopolysaccharide. Furthermore, intradermal injection of BPSV-CBP into injured skin, which more closely mimics BPSV lesions, delayed the influx of neutrophils and reduced the recruitment of MHC-II^+^ immune cells to the wound bed. Our findings suggest that the CBP could be important in pathogenesis of BPSV infections.

## Introduction

Bovine papular stomatitis virus (BPSV) is classified as a species within the *Parapoxvirus* (PPV) genus of the family *Poxviridae* [1] and induces acute pustular lesions on the muzzle, lips, oral mucosa and occasionally teats of cattle, in particular calves. The pathology of BPSV infection is characterised by proliferative and erosive dermatitis, vacuolation and swelling of keratinocytes in the stratum spinosum, reticular degeneration and marked epidermal proliferation. Neutrophils migrate into areas of reticular degeneration and form microabscesses that subsequently rupture on the surface, giving rise to the pustular nature of the disease [2,3]. In the mild form of the disease, lesions are flat and typical inflammatory rings containing moderate infiltration of inflammatory cells have been observed around the involved areas [4,5]. Normally, the disease is non-systemic; however the virus has been detected in blood and lymphatic tissues of immunosuppressed animals [6,7]. The clinical course of the infection lasts several weeks and the virus can occasionally be transmitted to humans [2,4].

PPVs are emerging as masters at manipulating the highly-tuned immune environment of skin [8]. Previous studies of PPVs, in particular the type species orf virus (ORFV), revealed they have evolved a range of mechanisms that temporarily suppress inflammation within the infected site and delay the host’s immune response. These anti-inflammatory mechanisms include the secretion of a viral homolog of interleukin-10 that reduces the production of pro-inflammatory cytokines [9–16], and also secretion of a chemokine-binding protein (CBP) that impairs the chemokine network [14,17–21]. In the case of BPSV, this combination of secreted viral immunomodulators in conjunction with other virulence factors may be associated with frequent subclinical infections, reinfections, and virus persistence in its natural host [6,7,22,23].

Chemokines are a large family of small signalling proteins with multiple functions, including activation and attraction of leukocytes towards sites of inflammation or infection. Chemokines attach to the extracellular glycosaminoglycan (GAG) matrix with low affinity, and create a concentration gradient to direct trafficking of various immune cells across the endothelium to sites of inflammation or infection. Based on the presence and arrangement of the N-terminal cysteine residues, chemokines are divided into four groups; α (CXC), β (CC), γ (XC) and δ (CX_3_C) chemokines. Generally, α chemokines recruit neutrophils or lymphocytes, while β chemokines attract monocytes and T cell subpopulations. Both γ chemokines (lymphotactins) and δ chemokine (fracktalkine) are chemoattractant for T cells, NK cells and dendritic cells [24,25].

Recently we reported that the BPSV-CBP can target specific inflammatory chemokines and consequently suppress inflammation in a post-stroke treatment of the mouse brain [21]. Increases in plasma chemokines associated with stroke, in particular CCL2 and CXCL2 were blocked in mice treated with CBP and this was associated with reduced neurological deficit, fewer brain infiltrating leukocytes, and a smaller infarct compared with the control. Here, we describe the molecular and structural properties of BPSV-CBP, and show that it is able to bind a broad range of murine chemokines with high affinity, prevent neutrophil and monocyte migration using an *in vitro* chemotaxis assay and subsequently, inhibit inflammatory cell infiltration in murine skin in which inflammation was induced by intradermal injection with the bacterial antigen lipopolysaccharide. Further, we examined the ability of BPSV-CBP to regulate cell recruitment in a model of cutaneous wound healing and showed its ability to delay neutrophil and reduce MHC-II^+^ immune cell infiltration into wound skin.

## Materials and Methods

### Protein Expression and Molecular Analyses

The BPSV-CBP was expressed in 293-EBNA cells as a FLAG-fusion protein and purified by affinity chromatography as described previously [21]. The purified CBP was analysed by multi-angle laser-light scattering (MALLS) (Wyatt Technology) coupled with size-exclusion chromatography (SEC) [20]. To examine oligomeric states of BPSV-CBP, the protein was mixed with 2% 2-mercaptoethanol and boiled for 5 min before separation by SDS-PAGE (Mini-Protean^®^ III Electrophoresis, Bio-Rad) and staining with coomassie blue. For immunoblotting, proteins were transferred onto Hybond^TM^-C extra nitrocellulose membrane (Amersham Biosciences) and the FLAG-tagged CBP detected using HRP-conjugated anti-FLAG M2 monoclonal antibody (Sigma), and Super Signal^®^ West Pic Chemoluminescence Solution (Thermoscientific).

### Surface Plasmon Resonance (SPR) assay

Prior to SPR assay, the viral CBP was dialyzed in HBS-EP buffer (20 mM HEPES, 150 mM NaCl, 3.4 mM EDTA, 0.005% Polysorbate 20, pH 7.4) using 12–14 Da Spectra/Por^®^ 2 Standard Grade Regenerated Cellulose membrane (Spectrum Lab Inc.). All SPR experiments were performed at 25°C using a Biacore X100 instrument (Biacore) as described previously [21]. The CBP was immobilized on the surface of a CM5 sensor chip (Biacore) at ~500 response units (pg/mm^2^) in 10 mM sodium acetate pH 4.5 by standard amine coupling method. Recombinant mouse chemokines (R&D Systems, listed in Table 1) were reconstituted, and serial dilutions in HBS-EP buffer run over the CM5 chip in triplicate for 3 min. The chemokines were allowed to dissociate for 10 min and then the sensor chip was regenerated by injecting 10 mM Glycine pH 2.0 (GE Healthcare). The sensorgrams produced were globally fitted with a 1:1 binding model and used for kinetics analysis by BIAevaluation software (version 2.0.1 Biacore). To investigate GAG-binding potential of the BPSV-CBP, a serial dilution of heparin sodium salt (MW ~15 kDa, Sigma), as a model compound for GAGs, was passed across the sensor chip in a similar manner with chemokine testing. Moreover, to examine the interference of heparin with the CBP-chemokine binding, CCL2 (50 nM) was pre-incubated with increasing concentrations of heparin (25, 50, 250 and 500 nM) and tested by SPR assay as described previously [26, 27].

**Table 1:** A comparison of chemokine-binding profiles of BPSV-CBP and ORFV-CBP shows that both viral CBPs share broad-specificity chemokine-binding properties.

		BPSV-CBP	BPSV-CBP	ORFV-CBP	ORFV-CBP
Chemokines		ELISA [21]	SPR	ELISA [20]	SPR [18–20]
**α**	**CXCL1**	NT	+	NT	NT
	**CXCL2**	+	++	+	NT
	**CXCL4**	+	+	+	+
	**CXCL8**	-	NT	-	NT
	**CXCL10**	-	-	-	-
	**CXCL12**	-	-	-	NT
**β**	**CCL2**	+	++	+	++
	**CCL3**	+	++	++	++
	**CCL5**	+	+++	++	+++
	**CCL19**	++	+++	++	+++
	**CCL21**	NT	++	NT	+
	**CCL22**	NT	-	NT	NT
**γ**	**XCL1**	+	+	+	+
**δ**	**CX3CL1**	-	-	-	NT

+++ <100 pM

++ 100–1000 pM

+ >1 nM

### Sequence and structural analysis

CBP sequence of BPSV strain V660 (GenBank: KM400588.1) was aligned with ORFV-CBP stain NZ2 (GenBank: ABA00630.1) using a Fold and Function Assignment System (FFAS) server (available at http://ffas.sanfordburnham.org), and the analysis was visualised using Aline software [28]. To highlight conserved residues between CBPs of BPSV and ORFV, the chemokine CCL2/ORFV-CBP complex structure (PDB: 4ZK9) was used to map these residues using PyMOL.

### Cells and Transwell Migration Assay

Neutrophils were derived from MPRO cell line (Clone 2.1, ATCC^®^ CRL-11422™) cultured in IMDM (Gibco^®^) supplemented with 20% horse serum (Gibco) and 10 ng/ml GM-CSF (R&D sys.) [29]. MPRO differentiation was induced with 10 μM all-trans retinoic acid (Sigma) for 3 days, and morphologic maturation was confirmed by Giemsa May-Grünwald staining (Sigma) of cytopsins under light microscopy (data not shown).

Monocytes were generated from murine bone marrow as described previously by Francke *et al*. [30]. Briefly, 8- to 10-week old male C57Bl/6 mice were sacrificed, and femurs and tibias were excised. The bone marrow was extracted in complete medium that contained RPMI-1640 (Gibco) supplemented with 10% heat-inactivated FBS (Gibco) and M-CSF (10% L929 supernatant). The bone marrow cells were seeded at 3 x 10^5^ cells/ml in ultra-low attachment surface plates (Corning Costar) and incubated at 37°C with 5% CO_2_ (Panasonic). On day three, the cells were fed by adding fresh medium, and non-adherent cells were harvested on day five.

For FACS analysis, the cells (neutrophils and monocytes) were washed, blocked by anti-mouse CD16/CD32 (BD Biosciences), and stained with the following antibodies and their isotype controls; neutrophils: AF488-Ly6G (clone RB6-8C5, eBioscience), APC-CD11b (clone M1/70, BD Biosciences) and PE-CXCR2 (clone 242216, R&D Systems), monocytes: AF488-CD115 (AFS98, Biolegend), APC-CD11b (clone M1/70, BD Biosciences) and AF594-Gr-1 (RB6-8C5, Biolegend). 2 μl of 7-Amino-Actinomycin D (7AAD, BD Biosciences) was also added to each sample to exclude nonviable cells. Flow cytometry data were acquired from 10,000 events of each sample using a FACSFortessa (BD Biosciences) and analysed by FlowJo version 10 (Tree Star).

The transwell migration assays were performed using 6.5 mm-diameter Corning Transwell 24-well plates (Corning Life Sciences) with either 3 μm (for neutrophils) or 5 μm (for monocytes) pore size polycarbonate membranes. All transwell assays were performed in duplicate with control wells. Prior to each assay, transwell plates were pre-equilibrated with 1 ml transwell medium (growth medium with 1% BSA) at 37°C with 5% CO_2_ for 1 h. Neutrophils (5 x 10^5^) or monocytes (1 x 10^5^) suspended in 100 μl transwell medium were carefully placed into the inserts, and the plates were incubated at 37°C with 5% CO_2_ (Panasonic). The incubation times for neutrophils and monocytes transmigration were 2 and 3 h, respectively [21,28]. In neutrophil migration assays, transmigrated cells were collected from the lower chambers and counted simultaneously with a volume of 30 μl of AccouCount fluorescent particles (ACFP) (Spherotech) on a flow cytometer (FACSFortessa, BD) for 2 min [31]. In monocyte migration assays, the transmigrated cells were collected from the bottom well of each sample, stained with 0.4% trypan blue solution, and counted manually on a hemocytometer [32]. Since a number of migrated monocytes were adhered to the underside of the membrane of transwell inserts, those cells were also stained and enumerated [18].

### Inflammatory Skin Model

All the animals were obtained from the University of Otago Animal Facility and used with ethical approval (no.37/13) from the University of Otago Animal Ethics Committee. In a preliminary experiment, 8-weeks old female SPF C57BL/6 mice (n = 10) were administered with 1 μg lipopolysaccharide (LPS) in 20 μl phosphate-buffered saline (PBS) injected intradermally, and the immune cell influx was monitored over a 24 h time course as described previously [33]. Each mouse received two LPS injections and two PBS-only injections in the shaved skin of the abdominal region. Animals were sacrificed at five time points (0, 3, 6, 12 and 24 h post infection) and the skin samples were harvested from the marked injection sites using a 6-mm disposable skin biopsy punch (Kruuse). One skin sample per condition from each mouse was used to extract myeloperoxidase (MPO) in 0.5% HTAB (Sigma) according to an established method [34,35]. The supernatant was added to TMB substrate solution (BD Biosciences) in triplicate and the absorbance was measured spectrophotometrically at 450 nm (Biorad) as reported earlier [36]. The other skin sample of each condition was processed for histology as described earlier [37]. Serial sections from three zones with 50 μm intervals were prepared for triple immunofluorescent staining using AF594-Gr1 (clone RB6-8C5, Biolegend), FITC-MHC-II (Clone M5/114.15.2, Abcam^®^) and DAPI (Invitrogen). The representative sections were visualized under 20x magnification and photographed (Olympus BX-51). Gr-1^+^ MHC-II^+^ neutrophils in four fields of sections from three zones with 50 μm intervals were counted on CellSens platform (Olympus). To test the effects of CBP in the inflammatory skin model, mice were co-injected with LPS and different doses of the CBP and used for evaluation of inflammatory cells infiltration. The animals (total number = 36) were divided into three dose-groups (0.01, 1 and 100 ng CBP) and each animal received four intradermal injections including PBS-only, LPS-only, LPS plus CBP and CBP-only. The animals were sacrificed 12 h later and skin samples were taken and prepared as described above.

### Wound Skin Model

SPF female C57BL/6 mice (6–8 weeks of age) were obtained from the University of Otago Animal Facility and were used with institutional ethical approval (no. 67/08). Following anesthesia with ketamine/domitor/atropine (75/1/0.05 mg/kg body weight, respectively), mice received two full-thickness, 4-mm-diameter, punch wounds administered as previously described [37,38]. Upon homeostasis, treatment groups were administered CBP (1 μg in 50 μL PBS), or PBS alone by subcutaneous injection adjacent to the wound on each flank (8 mice, each with 2 wounds, per group). An additional group of 8 mice was left untreated. Bupivacaine (2 mg/kg body weight) was administered for pain relief and strepsin to prevent wound infection before mice were revived using Antisedan (Zoetis, 5 mg/kg body weight). Four mice from each group were euthanized after 24 h and 48 h. Wound biopsies (1 cm^2^) were taken, divided in half along the anterior-posterior axis, then were fixed in 0.5% zinc salts solution and processed into paraffin wax. A section (4 μm) from the middle of each wound then underwent immuno-fluorescent staining using the anti-Gr-1/Ly6G (RB6-8C5) Alexa Fluor® 488 conjugate (eBioscience) and anti-MHC-II (Clone M5/114.15.2) FITC conjugate (Abcam). The staining was visualized using a fluorescent microscope with digital photographs taken of the entire section. Images were subsequently merged and converted into panoramas using Adobe Photoshop. The area of the fibrin clot and the dermis and hypodermis of the wound edges (to a point 0.75 mm distal to the cut edges of the panniculus carnosus), and the area of Gr-1 staining within that region, were quantitated using Image J, and results are expressed as the mean percentage area of Gr-1 (Gr-1 area / combined area of the fibrin clot and wound edges X 100). The numbers of MHC-II^+^ cells within the same area were quantitated manually and results were expressed as the mean MHC-II^+^ cells per mm^2^.

## Results

### BPSV-CBP is a homodimer

**Fig 1:**
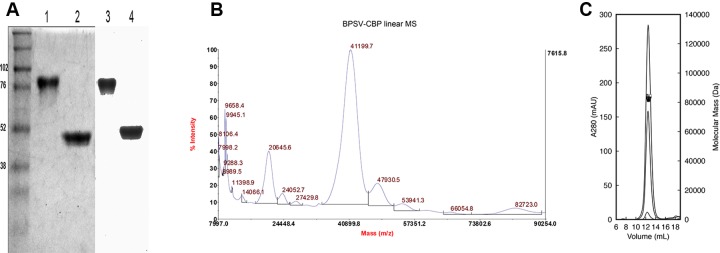
Molecular characterisation of BPSV-CBP. (A) Coomassie blue staining of SDS-PAGE (left) and Western Blot (right) analysis of the BPSV-CBP shows protein bands of ~80 kDa under non-reducing conditions (lanes 1 and 3) and ~45 kDa under reducing conditions (lanes 2 and 4). (B) MALDI tandem Time-of-Flight mass spectrometry analysis of the BPSV-CBP showing the main monomeric species has 41199.7 and 20645.9 m/z for 1+ and 2+ ions, respectively (MALDI TOF/TOF, Applied Biosystems). (C) Multi-angle laser-light scattering size-exclusion chromatography (MALLS-SEC) analysis of the BPSV-CBP over three concentrations (2, 1, 0.05 mg/ml) shows estimated molecular mass for the dimer protein is 82.4 kDa.

The CBP derived from BPSV was expressed in 293-EBNA cells and purified by affinity chromatography as described previously [21]. Analysis of BPSV-CBP by SDS-PAGE followed by Western Blot showed bands at ~80 kDa and ~45 kDa under non-reducing and reducing conditions, respectively (Fig 1A). In addition, the purified BPSV-CBP was analysed by mass spectrometry, and also multi-angle laser-light scattering size-exclusion chromatography (MALLS-SEC), which revealed that the CBP forms a homodimer with a molecular mass of 82.4 kDa in solution (Fig 1B and 1C). These findings are similar to the ORFV-CBP that exists as a homodimer [20]. The observation of protein bands larger than the theoretical size (MW = 32.97 kDa) can most likely be attributed to post-translation modifications such as glycosylation as shown for ORFV-CBP [20].

### BPSV-CBP is a broad-spectrum chemokine-binding protein

To assess the binding activity of the BPSV-CBP, it was immobilized on a sensor chip and the affinity and kinetics of thirteen murine chemokines were determined using an SPR assay (S1 Table). Among the α chemokines, fast association rate constants were observed for CXCL1, CXCL2 and CXCL4 interacting with the CBP (*k*_a_ = 0.7–1.0 x 10^6^ M^-1^s^-1^). However, CXCL1 and CXCL4 chemokines dissociated quickly (*k*_d_ = 2.3–3.2 x 10^−3^ s^-1^), resulting in a moderate binding affinity (*K*_D_ = 4.3 and 2.3 nM, respectively) while CXCL2 dissociated more slowly (0.6 x 10^−3^ s^-1^), thus presenting a stronger binding affinity in the picomolar range (*K*_D_ = 725 pM). All tested β chemokines, except for CCL22, bound the BPSV-CBP with fast to very fast association rate constants (*k*_a_ = 1–13 x 10^6^ M^-1^s^-1^). Chemokines CCL5 and CCL19 had extremely slow dissociation rate constants (*k*_d_ = 2 x 10^−5^ s^-1^) resulting in a very tight binding (*k*_D_ = 1.5–3.8 pM). The XCL1 chemokine also bound relatively strongly (*K*_D_ = 1.4 nM) with a fast association rate constant (*k*_a_ = 1 x 10^6^ M^-1^ s^-1^) and moderate dissociation rate constant (*k*_d_ = 1.4 x 10^−3^ s^-1^). No binding was observed using the CX_3_CL1 chemokine. Overall, the SPR probing indicated that the BPSV-CBP is able to bind a broad-spectrum of chemokines with significant affinity (S1 Fig).

The chemokine binding specificities determined by SPR in this study were compared with data obtained by an ELISA approach [21]. In general, there was a consistent correlation between the SPR and ELISA results (Table 1) indicating that the negative SPR results were not due to masking of binding sites of the CBP during the immobilization step. Minor differences between the results of the two methods might be related to different experimental conditions of the assays. Comparison of the chemokine-binding profile of BPSV-CBP with ORFV-CBP revealed that the BPSV-CBP shares similar broad-specificity chemokine-binding properties with ORFV-CBP (Table 1).

The ORFV-CBP data [18–20] and BPSV-CBP ELISA data [21] are adapted from previous studies. All chemokines are mouse origin except for human CCL2, CCL5, CXCL4, CXCL8 CXCL10, CXCL12 and XCL1 used in the ELISA assay. NT = not tested.

Previous SPR assays have shown that certain viral CBPs such as M-T1 of myxoma virus can directly bind GAG molecules [39], or like E163 and the SECRET domain of CrmD from ectromelia virus [27,40] and A41 of vaccinia virus [26] have capability to interfere with chemokine-GAG binding. The BPSV-CBP however, did not show any binding signal to heparin even at the highest concentration (100 μM) tested (S2A Fig). Moreover, in the competitive binding assay, heparin (even at CCL2 to heparin molar ratio 1:10) did not interfere with the BPSV-CBP binding to CCL2 (S2B Fig). This result indicates that there is no overlap between the chemokine-binding domain and GAG-binding domain of the BPSV-CBP, which is in agreement with the theoretical model predicted in the following section. Our results cannot be considered as conclusive proof that BPSV-CBP does not bind GAG and further studies will be carried out in the future.

### Structural properties of the BPSV-CBP

**Fig 2:**
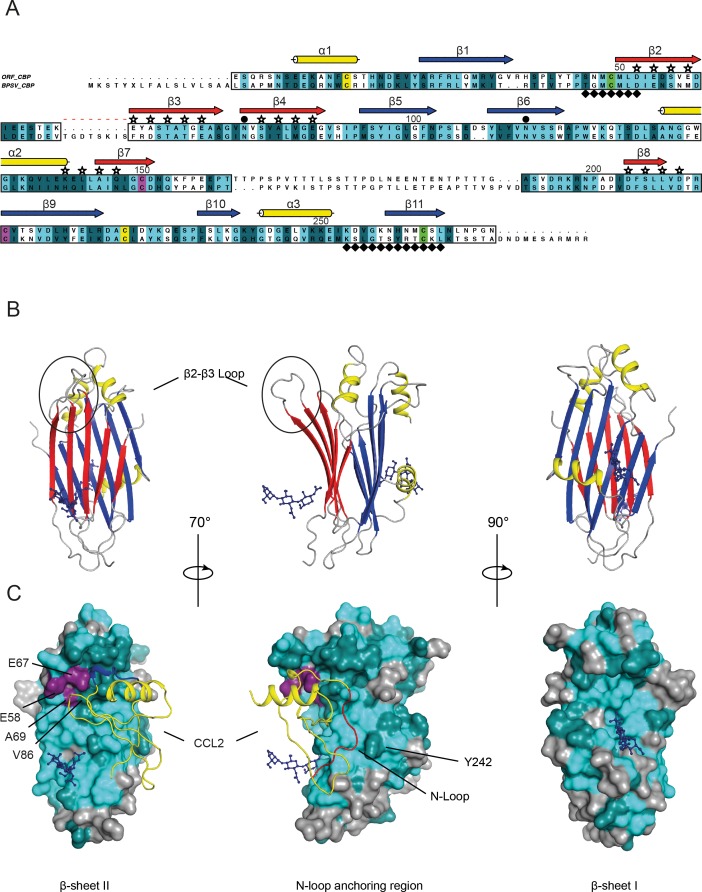
Structural analysis of BPSV-CBP. (A) Structure-based sequence alignment of BPSV-CBP and ORFV-CBP. Amino acid sequence of BPSV-CBP is compared with the sequence of ORFV-CBP represented in a published crystal structure (PDB: 4P5I). Identical and conserved residues are highlighted in light and dark cyan, respectively (definition of conserved amino acid residues follows ClustalW). The structurally conserved region (boxed) predicts BPSV-CBP has a common β-sheet sandwich structure including three α-helices (yellow cylinders) and two β-sheets (arrows). The β-sheet I (blue arrow) enriched with basic residues, while the β-sheet II (red arrow) comprises acidic and hydrophobic residues, and serves as chemokine-binding surface. The chemokine-binding residues exposed at the β-sheet II are indicated by stars. Six conserved cysteines forming disulphide bonds are highlighted in pair colours; yellow, green and purple. The N-linked glycosylation sites and dimerization interfaces are symbolized by ● and ♦, respectively. The red dashed line shows a distinct region in the connecting loop between β-strand 2 and 3 of BPSV-CBP. The numbering is based on the ORFV-CBP. (B) Cartoon representation of the ORFV-CBP monomer shows a typical β-sandwich fold. Glycosylation sites (dark blue ball and stick representation) are seen on both β-sheets. The major difference in the BPSV-CBP occurred in a connecting loop between β strands 2 and 3 (circle). The colour scheme for secondary structures is identical to Fig 2A. (C) Mapping of conserved residues of the BPSV-CBP onto the crystal structure of ORFV-CBP in complex with CCL2 (yellow ribbon diagram). The colour scheme of identical light and conserved dark cyan residues follows Fig 2A. A localized hydrophobic interaction pins-down the chemokine’s N-loop (red) into the CBP’s hydrophobic pocket (middle diagram), and the positively charged 20s loop of the chemokine (blue) sites within the negatively charged groove on the β-sheet II (left diagram). The altered amino acid residues on the β-sheet II (in purple) would not interfere with chemokine binding. Glycosylation sites are shown as dark blue ball and stick representation.

The structural-based sequence alignment of BPSV-CBP and ORFV-CBP showed 45.5% identity (107/235 residues) and 73.2% similarity (172/235 residues) over the core protein structure, supporting the comparable chemokine-binding specificity of the two CBPs (Fig 2). To gain further structural insights into the BPSV-CBP, we used the ORFV-CBP whose crystal structure alone and in complex with host chemokines have been solved recently [20]. Couñago *et al*. showed that the ORFV-CBP is a β-sheet sandwich composed of three α helices and two β-sheets; β-sheet I is positively charged, while β-sheet II is negatively charged and contains chemokine-binding sites. The chemokine-binding of the ORFV-CBP has been explained by interactions of the N-terminal signalling domain of chemokines with a hydrophobic pocket on the CBP, and also a polar interaction between the chemokine 20s loop with a broad negative groove on the β-sheet II surface [20].

Mapping the sequence analysis onto the ORFV-CBP crystal structure provided a clearer view of the likely chemokine-binding properties of BPSV-CBP. In the hydrophobic pocket, most of the interacting residues are identical and only Tyr242 is replaced with histidine. Also, the majority of the key residues exposed on the β-sheet II surface are conserved. However, a few major modifications were noticed; an insertion at the connecting loop between β strands 2 and 3 which leads to an extended loop region between two strands, and also two of the acidic residues (Glu58Met and Glu67Phe) and an alanine residue (Ala69Asp) that position proximal to this loop are changed, which potentially can affect electrostatic charge and thus binding affinity of the protein. Moreover, amino acid Val86 is replaced by methionine whose side chain is larger but maintains hydrophobicity. However, in the ORFV-CBP/CCL2 complex structure, the Val86 does not make direct contact with CCL2 and there is enough space to accommodate a methionine substitution in this structure. Nevertheless, as the overall geometry and surface charges of the protein are maintained, the negative influence of these modifications would be expected to be minimal and the structural analysis could explain the broad-spectrum chemokine-binding specificity of the BPSV-CBP. In addition, structural similarity between the CBPs of BPSV and ORFV is consistent with a recent study on structural analysis of poxvirus immune evasion proteins [41] where a conserved β-sandwich fold was identified among four groups of apparently-unrelated poxviral proteins, and identified them as members of a poxvirus immune evasion domain superfamily.

A previous study on the ORFV-CBP showed that it is a homodimeric glycoprotein. Dimerization takes place at β-strands 2 and 11, and doubles the interacting surface [20]. The structural analysis of the BPSV-CBP could explain its dimer arrangement found by MALLS-SEC analysis as the residues that aligned with the ORFV-CBP β-strand 2 are almost identical (6 out of 7). Moreover, like ORFV-CBP, the BPSV protein is also predicted to be glycosylated based on the existence of the conserved N-linked glycosylation sites (Fig 2A).

### BPSV-CBP inhibits neutrophil and monocyte chemotaxis *in vitro*

BPSV skin lesions are inflamed and contain a moderate infiltration of inflammatory immune cells such as neutrophils, macrophages and lymphocytes [2,3]. Neutrophils are among the first cells to respond to injury or infection and form the first line of defence against invading microorganisms. As the CBP expressed by BPSV binds the neutrophil chemoattractants CXCL1, CXCL2 and CCL3, we examined its ability to inhibit neutrophil chemotaxis using a transwell migration system. In addition we tested the ability of CBP to inhibit monocyte chemotaxis to the chemoattractants CCL2, CCL3 and CCL5.

**Fig 3:**
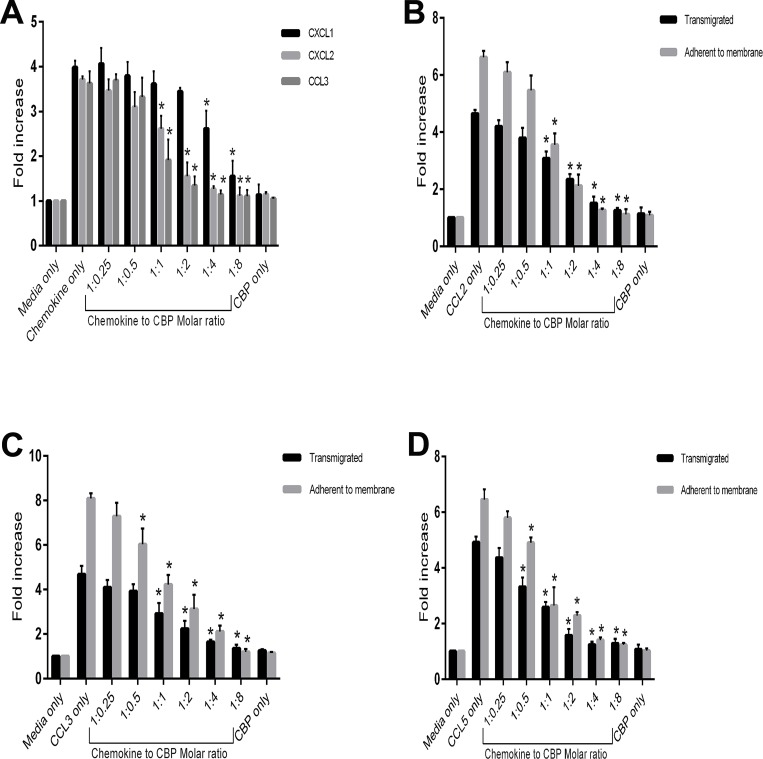
Inhibitory effect of the BPSV-CBP on *in vitro* migration of neutrophils and monocytes in response to CC and CXC chemokines. (A) 1 x 10^5^ neutrophils were placed into the upper chamber of the transwell assay system containing CXCL1, CXCL2 (200 ng/ml) or CCL3 (100 ng/ml) plus serial dilutions of the CBP to give the molar ratios shown (chemokine to CBP), and incubated for 2 h. The migrated cells were collected and counted by flow cytometry. The fold increase represents the migration of neutrophils compared with the media-only control. The combined data are shown as mean ± SD of three independent experiments conducted in duplicate and significant differences to chemokine-only are indicated by asterisks (*P* < 0.05, Tukey’s test analysis of variance, (ANOVA) GraphPad Prism). (B-D) 5 x 10^5^ monocytes were placed into the upper chamber of the transwell assay system containing CCL2 (50 ng/ml) (B), CCL3 (50 ng/ml) (C) or CCL5 (25 ng/ml) (D) plus serial dilutions of the CBP to give the molar ratios shown (chemokine to CBP), and incubated for 3 h. The transmigrated cells were collected and counted manually (black bars). The cells that adhered to the underside of the membrane were also stained and counted (grey bars). The fold increase represents the migration of monocytes compared with the media-only control. The combined data are shown as mean ± SD of three independent experiments conducted in duplicate and significant differences to chemokine-only are indicated by asterisks (*P* < 0.05, Tukey’s test ANOVA, GraphPad Prism).

Neutrophils were derived from a differentiated MPRO cell line that has been reported as a valid model for functional activity of murine neutrophils [29]. Morphologic maturation of MPRO cells following stimulation with retinoic acid was confirmed by microscopic examination, and flow cytometric analysis confirmed the cells were Ly-6G^hi^ CD11b^hi^ cells with ~80% expressing CXCR2 (S3 Fig). The reported optimum doses of chemokines for neutrophil migration *in vitro* vary from 50 ng/ml [31,42] to 8 μg/ml [43] depending on cell numbers and state, membrane pore size, incubation time and the enumeration method utilized. In initial experiments of this study, serial dilutions of neutrophil-attractant chemokines were tested to determine the optimum concentration for maximum cell migration. The chemokines induced a 3–4 fold increase in cell migration at 200 ng/ml of CXCL1 and CXCL2, and 100 ng/ml of CCL3 (S4 Fig). After optimizing the neutrophil chemotaxis assays, the effect of the BPSV-CBP was investigated by adding a titration of CBP to an optimal amount of chemokine in the transwell migration assay. Counting the migrated cells showed that the CBP was able to prevent neutrophil migration in a dose-dependent manner (Fig 3A).

Monocytes were generated from mouse bone marrow cell culture, and characterized as CD115^+^ CD11b^+^ cells with ~75% Gr-1 positivity (S5 Fig) and used in transwell migration assays. In this assay the cells that either migrated to the lower chamber or that adhered to the underside of the membrane were counted. Initial experiments showed a 4–7 fold increase in monocyte migration at 50 ng/ml of CCL2 and CCL5, and 25 ng/ml of CCL3 (S4 Fig). These findings are in the range reported previously [18,44–46]. A titration of the BPSV-CBP added to the optimum dose of chemokine showed a decrease in monocyte migration in a dose-dependent manner for the three chemokines tested (Fig 3B and 3D).

### BPSV-CBP inhibits recruitment of inflammatory cells to the skin

**Fig 4:**
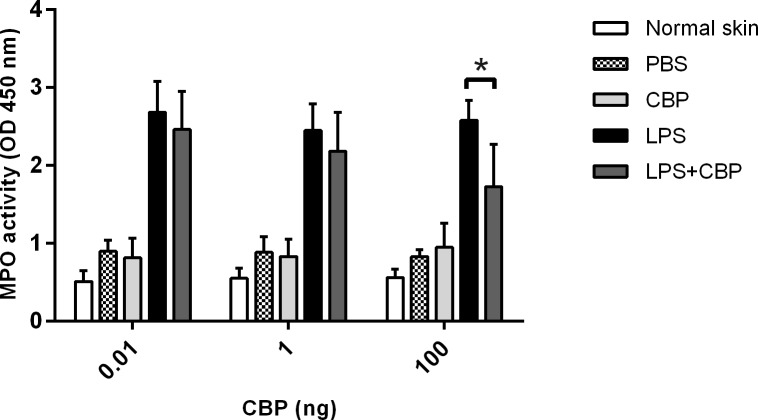
Myeloperoxidase analysis revealed that CBP blocks neutrophil infiltration in a murine skin inflammatory model. Twelve mice were divided into 3 groups of 4 and injected in the dermis with 1 μg LPS with and without the indicated amounts of CBP. In addition each mouse received injections of CBP only and PBS. i.e. a total of four injections per mouse. Each group received a different dose of CBP as shown above. At 12 h post-injection, mice were euthanized and 6 mm skin samples were taken and homogenized in HTAB to extract MPO. The harvested solution was added to TMB substrate in duplicate, and the enzyme activity was measured spectrophotometrically at 450 nm. The mean ± SD of the combined data of two independent experiments are shown and the asterisk indicates a significant difference (*P* < 0.05, Tukey’s test ANOVA, GraphPad Prism).

**Fig 5:**
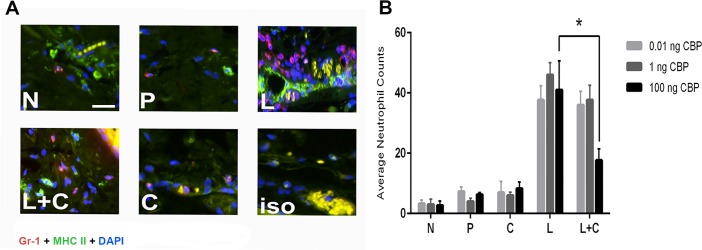
Histological analysis showed that CBP inhibited neutrophil influx in an inflammatory skin model. Twelve mice were injected in the dermis with 1 μg LPS with and without various amounts of CBP. In addition each mouse received injections of CBP only and PBS. i.e. a total of four injections per mouse (n = 4 animals per group for each dose of CBP). Mice were sacrificed at 12 h post treatment and skin samples were fixed in zinc salts solution and paraffin-embedded. (A) Skin sections were stained for neutrophils with triple immunofluorescent antibodies (AF594-GR1, FITC-MHC-II and DAPI) and visualised by fluorescent microscopy with 20x magnification. Scale bar = 20 μm. N = normal skin, P = PBS, L = LPS, L+C = LPS + CBP, C = CBP, iso = isotype control. (B) Numbers of Gr-1^+^ MHC-II^+^ neutrophils in four fields of sections from three zones with 50 μm intervals were enumerated. The data shown are the mean ± SD and the significant difference is indicated by the asterisk (*P* < 0.05, Tukey’s test ANOVA, GraphPad Prism).

To determine if our *in vitro* findings translated into inhibition of the recruitment of inflammatory cells during acute inflammation *in vivo*, a murine model of skin inflammation was used. Skin inflammation, induced with LPS, results in the recruitment of many different types of inflammatory cells to the injection site, including neutrophils [47,48], monocytes, dendritic cells and mast cells [18,19,49]. We investigated the effects of CBP on the infiltration of neutrophils as they are amongst the first cells recruited from the blood to reach the site of infection in relatively large numbers and are critical for driving the inflammatory response *via* expression of inflammatory cytokines. Furthermore, the chemokine-binding spectrum of BPSV-CBP and transwell functional data suggested that the CBP would inhibit the recruitment of these cells. Myeloperoxidase (MPO) is most abundantly produced by neutrophil granulocytes, and quantifying MPO activity is a well-established and sensitive method to measure neutrophil accumulation in inflamed tissue [50]. Even small injuries caused by control injections were reflected as a slightly higher level of MPO compared to the normal skin samples. It has been reported that intradermal injection of 1 μg of LPS causes a massive neutrophil swarm in mouse skin [47,48] and preliminary experiments of this study showed that LPS injection resulted in a 2–3 fold increase of MPO activity and localized skin inflammation by 12 h post injection (S6 Fig). Mice were then co-injected with LPS and different doses of the CBP (0.01, 1 and 100 ng) to investigate the effect of this viral protein on neutrophil infiltration. The MPO assay revealed that adding CBP can reduce neutrophil accumulation in response to LPS injection, however only at the highest dose of CBP (100 ng) was the reduction statistically significant (*P* < 0.05) (Fig 4). Histological examination confirmed there was a reduction in infiltrating Gr-1^+^ MHC-II^+^ cells that morphologically resembled neutrophils, into LPS-injected skin when 100 ng of CBP was co-administered (Fig 5). Furthermore, co-injection of LPS with 100 ng CBP caused a significant reduction in infiltration of MHC-II positive mononuclear cells that are collectively considered as inflammatory monocyte/DC population (S7 Fig).

**Fig 6:**
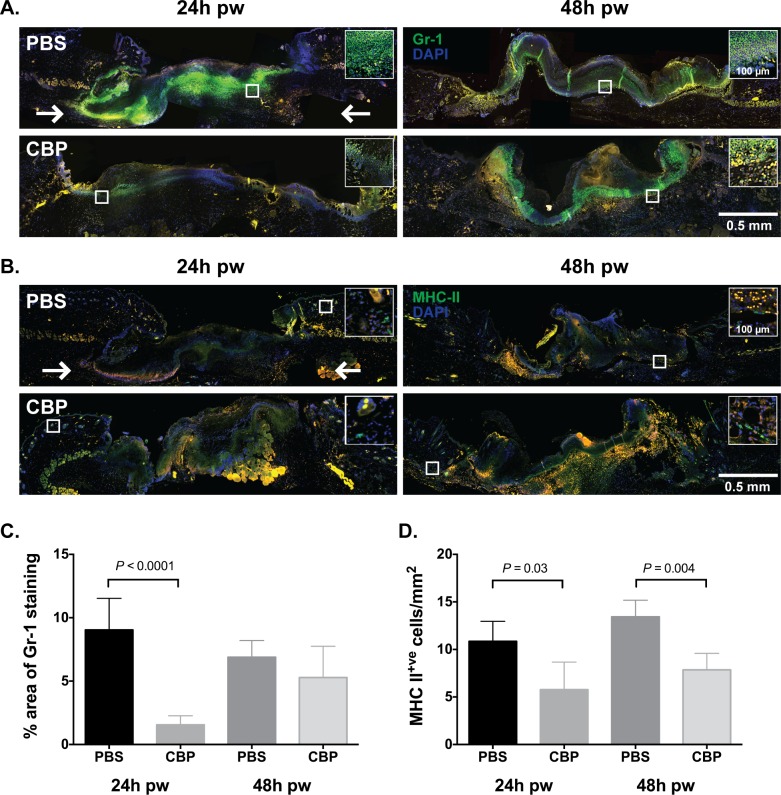
CBP delays neutrophil and MHC-II^+^ cell recruitment into wounded skin. Representative images showing the influx of neutrophils (A) and MHC-II^+^ cells (B) into wounded skin following injection of 1 μg CBP (CBP), 20 μl PBS (PBS). Skin biopsies taken 24 h and 48 h post wounding (pw) were fixed in zinc salts solution and paraffin-embedded. Four micrometer sections were stained for the neutrophil marker Gr-1 (green) and the nuclear stain DAPI (blue) or MHC-II (green) and DAPI. The wound bed and adjacent wound edges are shown and the location of the white box indicates the region of wounds. Two arrows located 0.75 mm distal to the cut edges of the panniculus carnosus indicate the points between which the area of staining was quantified. The relative area of Gr-1 staining (C) and number of MHC-II+ cells (D) at 24 h and 48 h was quantified in 1 section, from the region of greatest wound width, from 2 wounds from each of 4 mice per treatment and time point. Values are expressed as the percentage area of Gr-1 staining or MHC-II^+^ cell/mm^2^ ± SE. Significant points of difference between wound treatments (*P* < 0.05), as determined using ANOVA and the Bonferroni method, are indicated.

Our findings to date showed that the CBP from BPSV could reduce infiltration of inflammatory cells to the skin exposed to a bacterial antigen. BPSV infection however usually establishes in damaged skin, so we hypothesized that the CBP may be more effective against immune cells trafficking in a wound-like environment. We therefore set out to examine the ability of CBP to regulate Gr-1^+^ and MHC-II^+^ immune cells in a mouse model of cutaneous wound healing [37,38]. Excisional wounds were treated following homeostasis by subcutaneous injection of the CBP or PBS. After 24 or 48 h, the skin surrounding each wound was excised and the inflammatory cell content of the treated and untreated wounds were examined using immunofluorescent staining. After wounding, there was a dramatic increase in Gr-1 staining and MHC-II^+^ staining within wounded skin mock-treated by injection with PBS (Fig 6). Injection of CBP however, resulted in a significant 8-fold reduction in the area of neutrophil staining within the wound bed after 24 h (Fig 6C, *P* < 0.0001) and halved the number of MHC-II^+^ cells/mm^2^ at the wound edge compared with mock treated wounds (Fig 6D, *P* < 0.05). By 48 h however, the area of neutrophil staining within wounded skin treated with the CBP was equivalent to that of the control wounds (Fig 6C). However a large number of Gr-1^+^ cells were observed in the underlying dermal tissue in CBP-treated wounds, while in the control wounds, the Gr-1^+^ cells were localized within the fibrin clot. In contrast, the number of MHC-II^+^ cells had not increased by 48 h, and in both the mock and CBP-treated wounds, the MHC^+^ cells were penetrating into the wound bed (Fig 6D). Together these findings indicate that a single administration of the CBP delayed the migration of neutrophils and dampened MHC-II^+^ cell recruitment into wound skin.

## Discussion

This study characterised the molecular and biological properties of the BPSV-CBP that is divergent at the amino acid level from its counterparts in the *Parapoxvirus* genus. The most striking feature of the BPSV-CBP was its ability to bind a broad-spectrum of chemokines. The BPSV-CBP bound a range of inflammatory chemokines associated with the recruitment of monocytes and DCs to the sites of inflammation in skin whilst it also bound the constitutive chemokines that have a role in the trafficking of antigen presenting cells through lymphoid tissue. In addition, BPSV-CBP bound several CXC chemokines that are associated with the recruitment of neutrophils into inflamed skin and the XCL1 chemokine lymphotactin that attracts T cells. The structural analysis of BPSV-CBP based on the recently-published 3D structure of ORFV-CBP in complex with chemokines [20], provided molecular insights into its broad-spectrum chemokine binding properties. The two structures are very similar, and the critical amino acids present within the predicted chemokine-binding domain and the β-sandwich fold that provide the electrostatic and steric complementarity in ORFV-CBP, are conserved in BPSV-CBP. Furthermore, both BPSV-CBP and ORFV-CBP are dimers in solution. It is still unclear whether dimerization is functionally important for the biological activity of these CBPs however it is a marked difference to other poxvirus CBPs that have not been shown to form biologically relevant dimers. It is notable that CBP of certain herpesviruses, like murine γ-herpesvirus68, are also dimers in solution and this structural conformation is reported to be essential for their binding activity [51].

The PPV-CBPs are most closely related to the CBPs encoded by *Orthopoxvirus*es and *Leporipoxviruses*, known as type II CBPs, viral CC-chemokine inhibitors (vCCI), or the T1/ 35 kDa family of CC-CBPs that only target CC chemokines and/or GAG molecules, whilst the emerging picture from this study and previous studies on ORFV-CBP suggests that the PPV-CBPs have a broader chemokine-binding spectrum, and bind CXC and XC as well as CC chemokines [17–21]. The PPV-CBPs show a clear evolutionary divergence in both structure and chemokine-binding specificity compared with the vCCIs although their remains sufficient similarity in their structures to suggest a common ancestor. Broad-spectrum CBPs are not limited to PPVs and have been identified in some herpesviruses. For instance, Glycoprotein G (gG) of α-herpesviruses [52,53] and M3 of γ-herpesvirus [54,55] have been reported to bind a broad-spectrum of chemokines from the CXC, CC and XC groups. In addition M3 binds to CX3CL1.

Many types of immune cells are recruited into inflamed skin such as monocytes, dendritic cells, NK cells, mast cells, T lymphocytes and neutrophils. All these cells have a role in host defence against viruses. We have previously shown that ORFV-CBP potently blocks the recruitment of monocytes and dendritic cells in skin inflammation models that use CC chemokine gradients, so it was of interest to investigate whether or not the BPSV-CBP that binds the neutrophil-attractants CXCL1, CXCL2 and CCL3, had the capability to block the recruitment of neutrophils to the site of skin inflammation. Recent studies have changed the conventional view of neutrophils and showed that they may have a much wider role in developing immune responses than previously appreciated. Neutrophils are rapidly recruited from the blood to infection sites to form the first line of defence against invading microorganisms. Furthermore, they are critical for driving the inflammatory response by the secretion of IL-1β and TNF-α, and are now thought to be involved in linking innate and adaptive immunity [56]. In addition, it is now becoming clear that these cells have a role in host defence against viruses. They are crucial for NK cell homeostasis and function, a major cell population involved in the elimination of viruses [57,58]. Neutrophils also produce small peptides called defensins and some of these have recently been shown to have anti-viral activity against such viruses as herpes virus 1 and 2, vesicular stomatitis virus, influenza virus and adenovirus [59]. Moreover, neutrophils have been shown to release extracellular traps (“sticky” DNA threads) that are involved in defence against human immunodeficiency virus-1 [60], myxoma virus [61] and influenza virus [62,63]. Whether any of these mechanisms are effective against BPSV infection is not known. However what is clear is that the BPSV-CBP is effective at blocking the infiltration of these cells into the site of inflammation as is shown in the skin inflammation model and the wound model of this study. In the wound model, the CBP blocked the infiltration of neutrophils at day 1 pi by 80%. Interestingly the response was short lived and by 48 h post-injection there was no significant difference between the PBS control and where CBP was injected. This delay in the infiltration of neutrophils may be similar to that observed in a natural infection. As the disease develops, there are large numbers of neutrophils found within the lesion.

The finding that the PPVs have evolved a broad-spectrum chemokine binding protein, suggests that many types of cells are important in the hosts defences against these viruses such as monocytes, dendritic cells, NK cells, mast cells and phagocytic cells. In previous studies we have shown that ORFV-CBP is a potent inhibitor of monocytes and DCs in the mouse skin inflammation model [18,19]. In addition, we showed that ORFV-CBP blocked the migration of *ex vivo* CpG-activated DC to inguinal lymph nodes and T cell responsiveness [19]. We hypothesise that this response was due to the ability of the ORFV-CBP to bind the constitutive chemokines CCL19 and CCL21 that are critical for the trafficking of mature DC from the skin to peripheral lymph nodes. The results suggested that ORFV-CBP could potentially impair inflammation in the infected host in conjunction with other viral encoded immune modulators but also suppress the development of adaptive immunity. Given that the ORFV-CBP and BPSV-CBP have an identical chemokine-binding spectrum with high affinity for specific chemokines that recruit monocyte and DC (MHC-II^+^ cells) into inflamed skin, it would be reasonable to speculate that they will have similar roles in blocking cell trafficking in the infected host. Such effects can only be tested in the natural host by infection with gene deletion knock-out viruses. We have previously speculated that a likely role of orf virus CBP in conjunction with other secreted immunomodulators, is to delay the development of the adaptive immune response and this is partly supported by the observation that animals can be re-infected with the ORFV [10,64,65] although a memory response exists [8]. Similarly, BPSV is also known to re-infect its host [23,65,66] suggesting that the host adaptive response is impaired or that the development of the acquired response is delayed and it is possible that BPSV-CBP plays a role in this phenomenon.

It is noteworthy that other poxviruses that produce vCCIs also sequester a wide range of chemokines from different classes through such molecules as viral homologues of tumour necrosis factor (TNF) receptors that bind CC and CXC chemokines [27,67], or the interferon (IFN)-γ receptor that binds CC, CXC and C chemokine families through their conserved heparin binding domain [68]. Since such molecules are not encoded by PPVs, and the replication of the PPVs is limited to the skin epidermis or mucosal epithelium, it is apparent that these viruses have evolved additional chemokine-binding capability to survive and replicate in skin. Skin makes up the largest organ of the body and is arguably the most susceptible to infection since a break to the skin could allow microorganisms to enter the host. It is apparent that the skin immune system has evolved to rapidly respond to microbial invasion and it would appear that due to the narrow tissue tropism of the PPVs, they have evolved mechanisms to counteract such a potent inflammatory and immune response. The CBP in conjunction with other viral virulence factors, will most likely dampen the inflammatory response, shield virus-infected cells, and delay the development of the adaptive immune responses. Furthermore, this immune evasion strategy may explain persistent and subclinical BPSV infections [6,7,22,23] that have been reported previously.

Although inflammation is beneficial and necessary for host defence, unregulated activation of the immune system can cause serious tissue damage in various infectious and metabolic diseases [69,70]. Neutrophils dominate the first wave of immune cells emigrating into inflamed tissues, thus reducing excessive neutrophils could be a part of the treatment procedure in different inflammatory disorders. Neutrophil recruitment is precisely controlled by temporal and spatial cascades of multiple chemoattractants. Animal models suggest that these cascades are more vulnerable to inhibition in their early phases, as in later phases of inflammation the recruitment of neutrophils becomes reinforced by multiple overlapping pathways [71]. Therefore, antagonists to key neutrophil chemoattractants, like viral CBPs, that could impair their accumulation in early phases, may have therapeutic value for treatment of inflammatory conditions. However, neutrophil migration in response to chemokines is a complex reaction; they preferentially migrate toward the more distant attractant source [72] and reversibly lock on and off different chemoattractant signals under opposing chemoattractant gradients (oscillatory motion) [73]. Moreover, chemokines are at the “intermediate” level of chemotactic molecular hierarchy, and neutrophils eventually respond to “end-target” chemoattractants (e.g. bacterial products and component C5a) [70]. This phenomenon was observed in this study where the CBP delayed neutrophil infiltration in the wound model for 24 h and also in a previous study that showed post-stroke administration of the CBP reduced brain-infiltrating leukocytes, but only within the first 24 h [21]. These findings indicate that the BPSV-CBP is an attractive candidate for a virus-derived anti-inflammatory agent however, as the BPSV encodes an arsenal of weapons to suppress the host’s immune response, such a therapeutic agent should be combined with other key chemoattractant cues to control inflammatory cell trafficking and excessive inflammation in skin pathologies.

## Supporting Information

S1 Fig SPR sensorgrams illustrating the interactions between BPSV-CBP and mouse chemokines. Serial concentrations of the chemokines were injected in triplicate over the immobilized BPSV-CBP on a CM5 chip for 180 s, and then allowed to dissociate over 600 s. The obtained curves were globally fitted with BIAevaluation 3.2 software using a 1:1 binding model, and used for kinetics analysis presented in S1 Table. The result shows high affinity bindings with CC and CXC chemokines and no significant interaction with CX3CL1
(TIF)

S2 Fig SPR analysis of heparin binding to BPSV-CBP. (A) SPR sensorgram illustrating the interaction between BPSV-CBP and heparin as a model compound for GAG molecules. Serial dilutions (100 mM—100 μM) of heparin sodium salt (MW ~15 kDa, Sigma) was injected in triplicate over the immobilized BPSV-CBP on a CM5 chip for 180 s, and then allowed to dissociate over 600 s. No response was observed even using high concentration of heparin. (B) The SPR analysis of the interaction between BPSV-CBP and CCL2 chemokine in the presence of heparin. Mouse CCL2 (50 nM) was pre-incubated with increasing concentrations of heparin (25, 50, 250 and 500 nM) and then tested along with CCL2-only and heparin-only samples by the SPR assay as described above. The results shows that heparin (even at CCL2 to heparin molar ratio 1:10) does not decrease the response level indicating that there is no overlap between the chemokine-binding domain and GAG-binding domain of the BPSV-CBP.
(TIF)

S3 Fig FACS characterization of neutrophils. Neutrophils were derived from MPRO cell line with 10 μM all-trans retinoic acid for 3 days. More than 95% of the gated cells were alive (7-AAD negative) and express Ly6G and CD11b surface markers. Mature neutrophils are Ly-6G^hi^ CD11b^hi^ cells which express chemokine receptor CXCR2.
(TIF)

S4 Fig *In vitro* migration of neutrophils and monocytes in response to chemokines in transwell migration assays. Neutrophils (5 x 10^5^) (A) or monocytes (5 x 10^5^) (B-D) were placed into the top inserts of Transwell Permeable Supports containing serial dilutions of chemokines in the bottom wells. The neutrophil transwell plates were incubated for 2 h and migrated cells were collected and counted using flow cytometry. The monocytes transwell plates were incubated for 3 h, and transmigrated cells as well cell adhered on the bottom side of the membrane were counted and the fold increase was shown as grey and black lines, respectively. The data are shown as mean ± SD of three independent experiments in duplicates.
(TIF)

S5 Fig FACS characterization of monocytes. Monocytes were cultured from mouse bone marrow for five days. More than 95% of the gated cells were alive (7-AAD negative) and express monocytes general marker CD115 (M-CSF receptor). The monocytes are also positive for CD11b (96%) and Gr-1 (72%) surface markers.
(TIFF)

S6 Fig Preliminary evaluation of MPO activity in the inflammatory skin model. Ten mice which received intradermal injections of 1 μg LPS and PBS were sacrificed at five time points and skin samples were taken and frozen immediately. The MPO was extracted from homogenized skin samples in HTAB, and the enzymatic activity was measured spectrophotometrically in the presence of TMB substrate in duplicates. The asterisks indicate significant increase of MPO in injected sites compared with PBS controls at 6 h pi onward (*P* < 0.05, Tukey’s test ANOVA, GraphPad Prism).
(TIF)

S7 Fig The effect of BPSV-CBP on the recruitment of MHC-II^+^ immune cells in LPS-induced skin inflammation. Mice (n = 12) received intradermal injections of 1 μg LPS with and without various amounts of BPSV-CBP. Also each mouse received injections of BPSV-CBP only and PBS. Animals were sacrificed at 12 h post treatment and skin samples were stained with FITC-MHC-II and DAPI antibodies. Numbers of MHC-II+ cells in four fields of sections from three zones with 50 μm intervals were enumerated and shown as the mean ± SD. The results shows that co-injection of LPS with 100 ng BPSV-CBP induced a significant reduction in infiltration of MHC-II positive mononuclear cells that are collectively considered as inflammatory monocyte/DC population (*P* < 0.05, Tukey’s test ANOVA, GraphPad Prism). N = normal skin, P = PBS, L = LPS, L+C = LPS + CBP, C = CBP.
(TIF)

S1 Table Binding affinity and kinetics of the BPSV-CBP to murine chemokines tested by the SPR assay. (NB = no binding, NM = non-measurable binding).
(DOCX)
