# Supplementary figures and images for: A Broad-Spectrum Chemokine-Binding Protein of Bovine Papular Stomatitis Virus Inhibits Neutrophil and Monocyte Infiltration in Inflammatory and Wound Models of Mouse Skin
Source: PLoS One. 2016 Dec 9;11(12):e0168007. doi: 10.1371/journal.pone.0168007 (PMC5148066; doi:10.1371/journal.pone.0168007)

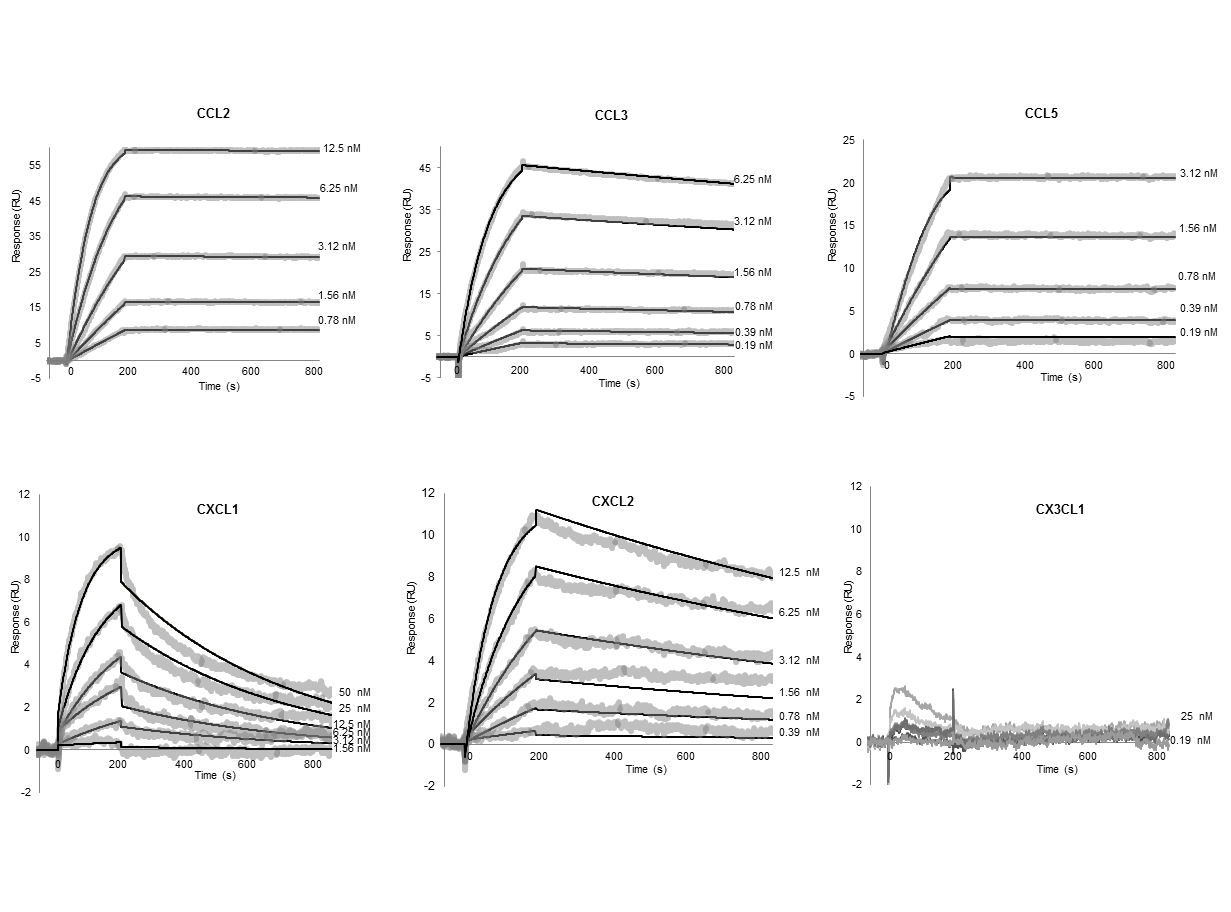

Supplement: S1 Fig — Serial concentrations of the chemokines were injected in triplicate over the immobilized BPSV-CBP on a CM5 chip for 180 s, and then allowed to dissociate over 600 s. The obtained curves were globally fitted with BIAevaluation 3.2 software using a 1:1 binding model, and used for kinetics analysis presented in S1 Table. The result shows high affinity bindings with CC and CXC chemokines and no significant interaction with CX3CL1 (TIF) [file pone.0168007.s001.tif]

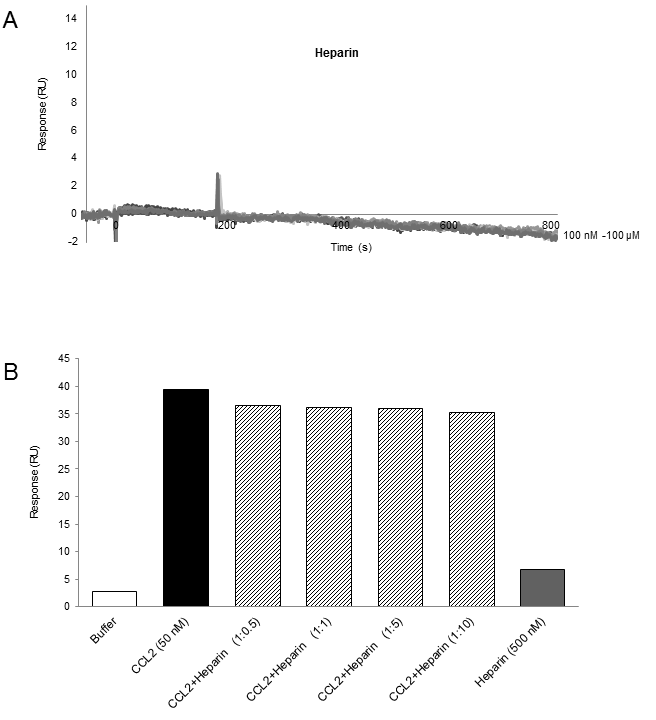

Supplement: S2 Fig — (A) SPR sensorgram illustrating the interaction between BPSV-CBP and heparin as a model compound for GAG molecules. Serial dilutions (100 mM—100 μM) of heparin sodium salt (MW ~15 kDa, Sigma) was injected in triplicate over the immobilized BPSV-CBP on a CM5 chip for 180 s, and then allowed to dissociate over 600 s. No response was observed even using high concentration of heparin. (B) The SPR analysis of the interaction between BPSV-CBP and CCL2 chemokine in the presence of heparin. Mouse CCL2 (50 nM) was pre-incubated with increasing concentrations of heparin (25, 50, 250 and 500 nM) and then tested along with CCL2-only and heparin-only samples by the SPR assay as described above. The results shows that heparin (even at CCL2 to heparin molar ratio 1:10) does not decrease the response level indicating that there is no overlap between the chemokine-binding domain and GAG-binding domain of the BPSV-CBP. (TIF) [file pone.0168007.s002.tif]

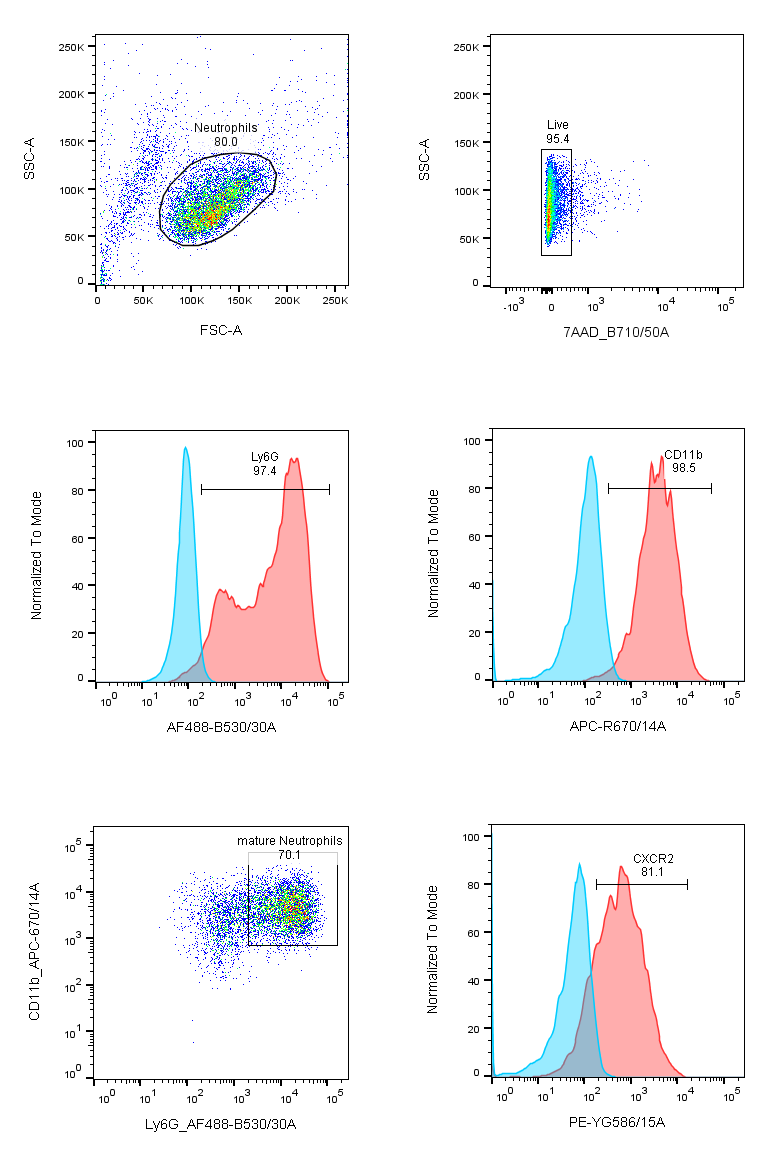

Supplement: S3 Fig — Neutrophils were derived from MPRO cell line with 10 μM all-trans retinoic acid for 3 days. More than 95% of the gated cells were alive (7-AAD negative) and express Ly6G and CD11b surface markers. Mature neutrophils are Ly-6Ghi CD11bhi cells which express chemokine receptor CXCR2. (TIF) [file pone.0168007.s003.tif]

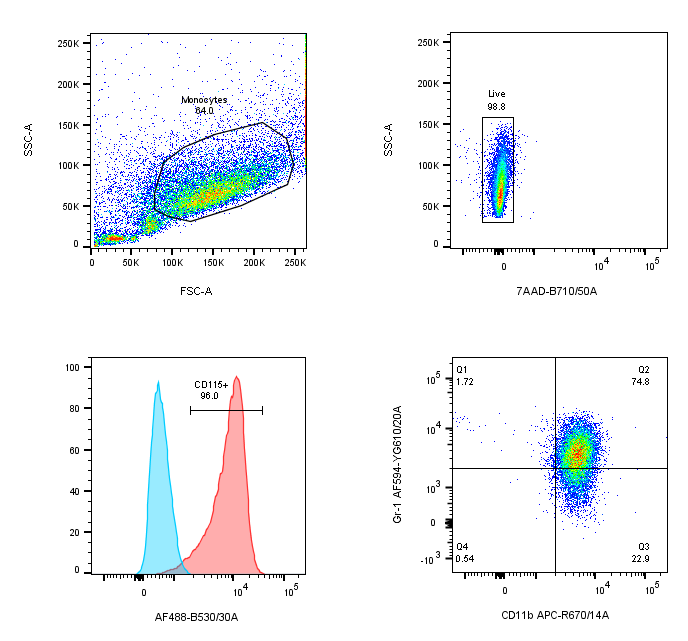

Supplement: S5 Fig — Monocytes were cultured from mouse bone marrow for five days. More than 95% of the gated cells were alive (7-AAD negative) and express monocytes general marker CD115 (M-CSF receptor). The monocytes are also positive for CD11b (96%) and Gr-1 (72%) surface markers. (TIFF) [file pone.0168007.s005.tiff]
