# Supplementary material for: A Broad-Spectrum Chemokine-Binding Protein of Bovine Papular Stomatitis Virus Inhibits Neutrophil and Monocyte Infiltration in Inflammatory and Wound Models of Mouse Skin
Source: PLoS One. 2016 Dec 9;11(12):e0168007. doi: 10.1371/journal.pone.0168007 (PMC5148066; doi:10.1371/journal.pone.0168007)
Supplement: S1 Table — (NB = no binding, NM = non-measurable binding). (DOCX) [file pone.0168007.s008.docx]

**S1 Table**

|  | **Chemokines** | ***k*_a_ (x 10^6^ M^-1^s^-1^)** | ***k*_d_ (x 10^-3^ s^-1^)** | ***K*_D_ (pM)** |
| --- | --- | --- | --- | --- |
| **α** | **CXCL1** | 0.73 ± 0.003 | 3.16 ± 0.02 | 4328 |
|  | **CXCL2** | 0.77 ± 0.004 | 0.56 ± 0.003 | 725 |
|  | **CXCL4** | 1.01 ± 0.03 | 2.34 ± 0.04 | 2316 |
|  | **CXCL10** | NM | NM | NM |
|  | **CXCL12** | NM | NM | NM |
| **β** | **CCL2** | 1.18 ± 0.04 | 0.22 ± 0.005 | 186 |
|  | **CCL3** | 1.76 ± 0.06 | 0.23 ± 0.005 | 130 |
|  | **CCL5** | 5.32 ± 0.09 | 0.02 ± 0.0004 | 3.75 |
|  | **CCL19** | 13.7 ± 0.9 | 0.02 ± 0.0003 | 1.46 |
|  | **CCL21** | 7.68 ± 0.1 | 2.96 ± 0.05 | 385 |
|  | **CCL22** | NM | NM | NM |
| **γ** | **XCL1** | 0.99 ± 0.02 | 1.35 ± 0.01 | 1358 |
| **δ** | **CX3CL1** | NB | NB | NB |
